# The 2R, 3R isomer of 2, 3-butanediol prevents weight gain and decreases plasma glucose concentration of mice fed a fat-enriched diet

**DOI:** 10.3389/fphys.2026.1721571

**Published:** 2026-05-25

**Authors:** Sunil Veeravalli, Toryn M. Poolman, Jeremy R. Everett, Ian R. Phillips, Elizabeth A. Shephard

**Affiliations:** 1 Department of Structural and Molecular Biology, University College London, London, United Kingdom; 2 Medway Metabonomics Research Group, University of Greenwich, Chatham Maritime, United Kingdom; 3 School of Biological and Behavioural Sciences, Queen Mary University of London, London, United Kingdom

**Keywords:** anti-obesogen, *Bifidobacterium*, flavin-containing monooxygenase, FMO5, gut microbiota, indolepropionate, liver metabolites

## Abstract

**Background:**

We have previously shown that, in comparison with wild-type mice, mice that lack a functional flavin-containing monooxygenase 5 gene (*Fmo5^-/-^* mice) exhibit reduced weight gain as they age, reduced fat deposition, and lower plasma concentrations of total cholesterol and glucose. We also identified isomers of 2, 3-butanediol as urinary biomarkers of *Fmo5^-/-^* mice. Treatment of male, wild-type mice fed a standard chow diet, with a 2, 3-butanediol isomer mix resulted in reduced epidydimal fat deposition and lower plasma cholesterol concentration. In the current study we explored the influence of individual isomers of 2, 3-butanediol on weight gain and plasma metabolite concentrations of wild-type mice fed a fat-enriched diet. We also investigated the effect of 2, 3-butanediol isomers on liver metabolites and gut microbiota.

**Methods:**

Mice were fed a standard chow or a 16% fat-enriched diet. Mice on the fat-enriched diet were untreated or treated with 2R, 3R-butanediol, 2S, 3S-butanediol, meso-2, 3-butanediol or a mix of the three isomers. Mice were analyzed for weight, plasma and liver metabolite concentrations and gut microbiota composition.

**Results:**

Treatment with 2R, 3R-butanediol prevented weight gain and decreased plasma glucose concentration of mice fed the fat-enriched diet. 2R, 3R-Butanediol increased the concentrations of three of the 693 liver metabolites identified: indolepropionate, *N*-delta-acetylornithine and 1-myristoyl-2-arachidonoyl-glycerophosphorylcholine (14:0/20:4). In comparison with untreated mice fed the fat-enriched diet, the gut microbiota of mice treated with 2R, 3R-butanediol was depleted in the Bacteroidota (Bacteroidetes) phylum and enriched in the Actinomycetota phylum, particularly in two species of *Bifidobacterium*, *B. globosum* and *B. 388775*.

**Conclusions:**

We have identified 2R, 3R-butanediol as an effective anti-obesogenic agent that prevents weight gain and decreases plasma glucose of mice fed a fat-enriched diet. The effect of 2R, 3R-butanediol may be due, in part, to its ability to increase the concentration of indolepropionate in the liver and of the proportion of *Bifidobacterium* species in the gut microbiota, both of which are known to have beneficial effects on weight control and plasma glucose concentration. Our results suggest that 2R, 3R-butanediol may be of value as a therapeutic agent to suppress weight gain in humans.

## Introduction

Flavin-containing monooxygenases (FMOs) of mammals are best known for their roles in the detoxification of foreign chemicals, including therapeutic drugs and dietary-derived compounds (Krueger and Williams, 2005; Phillips et al., 2007; Phillips and Shephard, 2017). However, through the use of knockout mouse lines, two members of this enzyme family, FMO1 and FMO5, are now known to have roles in endogenous metabolism (Phillips and Shephard, 2019). FMO1 is a regulator of energy balance (Veeravalli et al., 2014) and an enzyme that catalyzes the conversion of hypotaurine to taurine (Veeravalli et al., 2020). FMO5 is a regulator of metabolic aging (Gonzalez Malagon et al., 2015) and glucose homeostasis (Scott et al., 2017).

FMO5 is expressed primarily in the liver (Hernandez et al., 2004). It is also expressed in the stomach and in both the small and large intestine, where it is present in cells throughout the luminal lining (Scott et al., 2017), and plays a role in goblet cell maturation, mucus barrier formation (Scott et al., 2017; Schaller et al., 2025) and acts as a sensor of gut bacteria (Scott et al., 2017). Transcriptomic and metabolomic analyses of liver reveal that the protein influences a wide-range of metabolic processes (Phillips et al., 2023).

In comparison with wild-type mice, *Fmo5^-/-^* mice exhibit reduced weight gain as they age, reduced fat deposition, and lower plasma concentrations of total cholesterol and glucose (Gonzalez Malagon et al., 2015; Scott et al., 2017). Analysis of the urine of *Fmo5^-/-^* mice identified the presence of isomers of 2, 3-butanediol, namely meso-2, 3-butanediol (M) and enantiomeric 2R, 3R-butanediol (R) or 2S, 3S-butanediol (S). These molecules are absent from the urine of wild-type mice (Veeravalli et al., 2022) and thus are urinary biomarkers of *Fmo5^-/-^* mice. Antibiotic treatment markedly reduced the concentration of the 2, 3-butanediol isomers in the urine of *Fmo5^-/-^* mice, confirming the microbially dependent origin of the isomers (Veeravalli et al., 2022).

2, 3-Butanediol is the subject of much investigation because of its use in a wide range of applications in the agricultural, chemical, cosmetic and pharmaceutical industries (Ji et al., 2011; Song et al., 2019). The isomers of 2, 3-butanediol are produced by bacterial action, with different species favouring the production of different isomers (Song et al., 2019). Members of the *Bacillus* genus produce mainly the M and R isomers, whereas strains of *Klebsiella* and *Enterobacter* produce the S and M isomers. However, the production of the S enantiomer is rare (Ledingham and Neish, 1954). 2, 3-Butanediol is produced from pyruvate during microbial sugar fermentation (Ji et al., 2011). Microbial biosynthesis of 2, 3-butanediol, a neutral metabolite, is stimulated to prevent acidification by low-pH fermentation products (Bartowsky and Henschke, 2004; Van Houdt et al., 2007) and is reversibly transformed to acetoin, accompanied by the conversion of NAD to NADH. Therefore 2, 3-butanediol concentration is considered as playing a role in regulating the NAD/NADH ratio (Magee and Kosaric, 1987).

In a follow-up study to our findings on *Fmo5^-/-^* mice we investigated whether treatment with 2, 3-butanediol could influence fat deposition and plasma metabolites in wild-type mice (Veeravalli et al., 2022). Treatment of male, wild-type mice fed a standard chow (SC) diet, with a 2, 3-butanediol isomer mix representative of that observed in the urine of *Fmo5^-/-^* mice, resulted in reduced epidydimal fat deposition and lower plasma cholesterol concentration (Veeravalli et al., 2022).

In the current study we investigated the influence of each of the 2, 3-butanediol isomers on weight gain and plasma metabolite concentrations of wild-type mice fed a fat-enriched diet that reflects the fat content of a ‘Western’ diet. Individual isomers of 2, 3-butanediol were found to reduce or prevent weight gain of mice fed the fat-enriched diet. To gain insights into the underlying causes we investigated the effect of 2, 3-butanediol isomers on the concentrations of liver metabolites and the composition of gut microbiota.

## Materials and methods

### Animal maintenance and sample collection

Male wild-type C57BL/6J mice were bred at University College London (UCL) and housed in the same room and in adjacent cages. Mice were age-matched to ± 2 days. From weaning to 14 weeks of age mice were fed a standard chow (SC) diet (Teklad Global Rodent Diet, Cat No. 2018, 3.1 kcal/g, ENVIGO, Madison, WI, USA). At age 14 weeks, six cohorts were established. Cohort 1: mice (n = 10) were fed the SC diet, which contains 6% fat, for a further 17 weeks. Cohorts 2 – 6: mice were transferred to a 16% fat diet, (Teklad Custom Diet, Cat No. TD.96322, gamma irradiated, vacuum packed, 4.3 kcal/g, ENVIGO) for a further 17 weeks. The fat-enriched diet is termed high-fat diet (HFD) and was chosen because its fat content is thought to more closely resemble that present in the diet of many humans. After 10 weeks on the HFD, cohorts 3-6 (n = 10) were treated with isomers of 2, 3-butanediol for a further 7 weeks, while cohort 2 (n = 7) remained untreated. The dose of 2, 3-butanediol isomers was such that the isomer concentration in the urine of wild-type mice would be similar to that in the urine of untreated *Fmo5^-/-^* mice (Veeravalli et al., 2022). Mice were dosed orally in their drinking water; the dose being calculated based on the average daily volume of water intake. Cohort 3 (MRS group) was treated with an isomer mix, meso-2, 3-butanediol (Sigma-Aldrich), 2R, 3R-butanediol (Alfa-Aesar), 2S, 3S-butanediol (Fischer Scientific), in a 4:1:1 ratio (250 mg/kg/d); cohort 4 (M group) with the M isomer (168 mg/kg/d); cohort 5 (R group) with the R isomer (42 mg/kg/d); cohort 6 (S group) with the S isomer (42mg/kg/d). Mice were housed in adjacent cages, with free access to food and water. Consumption of water was measured twice weekly, and water was changed at these times. Food consumption and body weight were measured weekly. Fresh food was provided weekly. Animal procedures were approved by the UCL ethics committee (Animal Welfare and Ethical Review Body) and carried out under Home Office Licences in accordance with the UK Animal Scientific Procedures Act.

### Plasma analysis

Food was withdrawn for 12 hours before collection of blood samples. Blood samples were collected between 9:00 and 11:00 a.m. and plasma was isolated and analyzed at the Mary Lyon Centre at MRC Harwell, as described previously (Hough et al., 2002; Scott et al., 2017). Data are expressed as means ± SEM and were analyzed with an unpaired two-tailed t-test with Welch’s correction (statistical significance defined as a *p*-value of < 0.05). Statistical analysis was carried out using GraphPad Prism (version 10.4.1).

### Metabolomics

At the termination of the experiment, when mice were 31-weeks old, liver samples were collected between 9:00 and 11:00 a.m. Samples were immediately frozen on solid CO_2_ and stored at -80 °C until being shipped on solid CO_2_ to Metabolon (Morrisville, NC, USA) for global liver biochemical profiling. The Metabolon platform utilized Ultrahigh Performance Liquid Chromatography-Tandem Mass Spectroscopy (UPLC-MS/MS). Namely, a Waters ACQUITY ultra-performance liquid chromatography (UPLC) and a Thermo Scientific Q-Exactive high resolution/accurate mass spectrometer interfaced with a heated electrospray ionization (HESI-II) source and Orbitrap mass analyzer, operated at 35, 000 mass resolution. Following log transformation, Welch’s two-sample t-test was used to identify biochemicals that differed significantly, with an adjusted *p*-value (*q)* of *q* < 0.05, in abundance between each of the treated cohorts and the untreated HFD cohort. Details of the experimental methods and analytical procedures are given in **Supplementary Methods**. The biochemicals identified and *q*-values are given in **Supplementary Tables 1**–**4**.

### Analysis of gut microbiota

Fresh faecal samples were collected from 31-week-old individual mice at the termination of the experiment and stored at -80 °C until analysis. Although the faecal microbiota is not identical to that present in the entire gut, it is derived from the gut and reflects the composition of bacteria within the gut. Consequently, we use the term gut microbiota throughout. DNA sequencing was performed using primers against the V3-V4 region of the 16S rRNA gene by Novogene, Cambridge, UK. Fastq files were processed using the program Quantitative Insights into Microbial Ecology (QIIME2 v. 2023.9) (Bolyen et al., 2019). Initial quality plots were generated to select a truncation length (forward 225 bp, reverse 222 bp) to remove low quality reads. Primer sequences were removed using Cutadapt (Martin, 2011). Denoising was carried out using DADA2 (Callahan et al., 2016). Taxonomy was assigned using the Greengenes2 database (McDonald et al., 2024), a phylogenetic tree generated from aligned sequences using Fast-tree. QIIME2 output tables (qza files) were analyzed using QIIME2R. The phyloseq package (McMurdie and Holmes, 2013) was used to normalize the reads between the samples (rarefaction using a depth of 26264, corresponding to the sample with the lowest number of reads) and calculate alpha- and beta-diversity (with PERMANOVA). Alpha-diversity was analyzed by the use of observed features and Shannon index. Beta-diversity was analyzed using Bray-Curtis dissimilarity as displayed by principal coordinate analysis (PCoA). Linear discrimination analysis effect size (LEfSe) was carried out using the microbiomeMarker package (Segata et al., 2011; Cao et al., 2022).

## Results

### Treatment of mice with the R isomer of 2, 3-butanediol prevents weight gain on a fat-enriched diet

**Figure 1:**
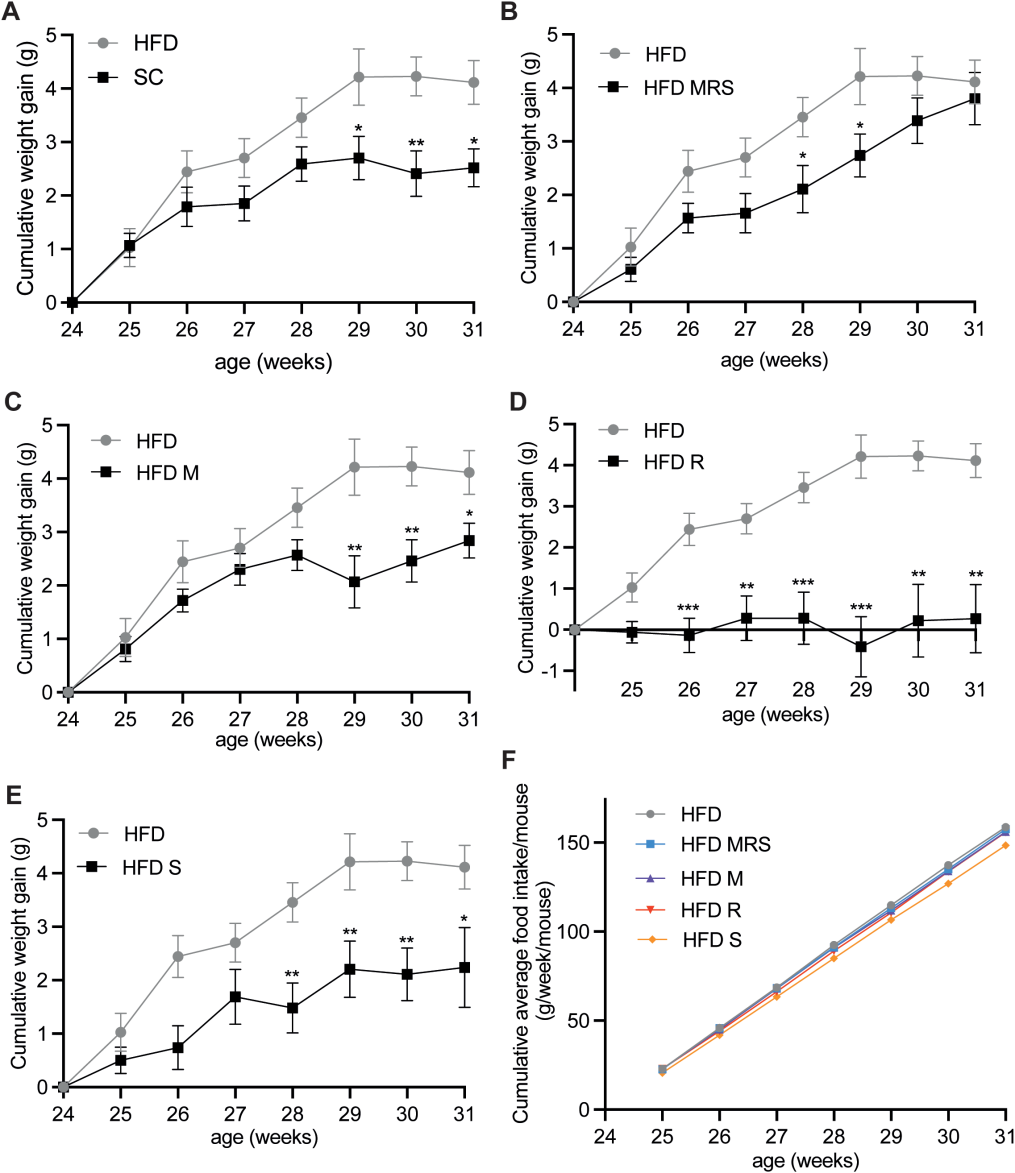
Influence of diet and 2, 3-butanediol isomers on weight gain and food intake. **(A)** Male mice were fed either a standard chow diet (SC) or a fat-enriched diet containing 16% fat (HFD). **(B–E)** Male mice were fed the HFD and were either untreated (HFD) or treated with 2, 3-butanediol isomer mix (HFD MRS) **(B)**, meso-2, 3-butanediol (HFD M) **(C)**, 2R, 3R-butanediol (HFD R) **(D)**, 2S, 3S-butanediol (HFD S) **(E)**. **(F)** Cumulative food intake of HFD-fed mice either untreated (HFD) or treated with the MRS (HFD MRS), M (HFD M), R (HFD R) or S (HFD S) isomers of butanediol. SC, n = 10, HFD untreated, n = 7, HFD-treated cohorts, n = 10. *p < 0.05, **p < 0.01, ***p < 0.001.

Mice fed the SC diet increased in weight, from 24 to 31 weeks of age, by 2.51 ± 0.75 g (**Figure 1A**), whereas mice fed for the same seven-week period on a 16% fat-enriched diet (HFD) that mimics a Western diet increased in weight by 4.11 ± 0.41 g (**Figure 1A**), resulting in a significant difference in weight between the two cohorts. Mice fed the HFD were treated with isomers of 2, 3-butanediol and their weight compared with that of untreated mice fed the HFD. Treatment of mice fed the HFD with a mix of MRS isomers of 2, 3-butanediol had some effect on decreasing cumulative weight gain, but by the end of the treatment period there was no significant difference in weight between treated and untreated animals (**Figure 1B**). In contrast, treatment of mice on the HFD with the M, R or S isomers of 2, 3-butanediol resulted in a significant decrease in cumulative weight gain (**Figures 1C–E**). In the case of the R isomer, weight gain was completely prevented (**Figure 1D**). There was no significant difference in cumulative average food intake among the different cohorts on the HFD (**Figure 1F**).

### Effect of treatment with isomers of 2, 3-butanediol on plasma constituents of mice fed a fat-enriched diet

Treatment of mice fed the HFD with the M, S or R isomers of 2, 3-butanediol, or with the MRS isomer mix, had no effect on plasma concentrations of total cholesterol or triacylglycerides (TAGs) (**Table 1**). However, treatment with either the R or S isomers of 2, 3-butanediol increased the plasma concentration of non-esterified fatty acids (NEFA), by 30%, and decreased that of glucose, by 22% (**Table 1**). The plasma concentration of glucose was also decreased, 16%, by treatment with the MRS isomer mix (**Table 1**).

**Table 1:** Effect of treatment with isomers of 2, 3-butanediol on plasma constituents of mice fed a fat-enriched diet.

	Untreated treated (n=7)	MRS treated (n=10)	M treated (n=10)	R treated (n=10)	S treated (n=10)
Total cholesterol (mmol/L)	4.80 ± 0.33	4.34 ± 0.19 *p* = 0.246	4.91 ± 0.47 *p* = 0.231	4.02 ± 0.26 *p* = 0.077	4.30 ± 0.40 *p* = 0.342
Triglycerides (mmol/L)	1.01 ± 0.04	1.05 ± 0.05 *p* = 0.559	1.05 ± 0.07 *p* = 0.598	1.10 ± 0.07 *p* = 0.299	1.10 ± 0.10 *p* = 0.420
NEFA (mmol/L)	1.05 ± 0.11	1.25 ± 0.08 *p* = 0.178	1.27 ± 0.10 *p* = 0.156	1.36 ± 0.07 *p* = 0.039	1.36 ± 0.04 *p* = 0.032
Glucose (mmol/L)	12.57 ± 0.56	10.57 ± 0.57 *p* = 0.025	11.77 ± 0.75 *p* = 0.402	9.85 ± 0.70 *p* = 0.009	9.76 ± 0.40 *p* = 0.002

Male mice were fed a fat-enriched diet containing 16% fat (HFD) and were untreated or treated with 2, 3-butanediol isomer mix (MRS treated), meso-2, 3-butanediol (M treated), 2R, 3R-butanediol (R treated) or 2S, 3S-butanediol (S treated). NEFA, non-esterified fatty acids; n = number of animals; *p-*values refer to comparisons between animals treated with 2, 3-butanediol and untreated animals.

### Effect of treatment with isomers of 2, 3-butanediol on liver metabolites of mice fed a fat-enriched diet

**Figure 2:**
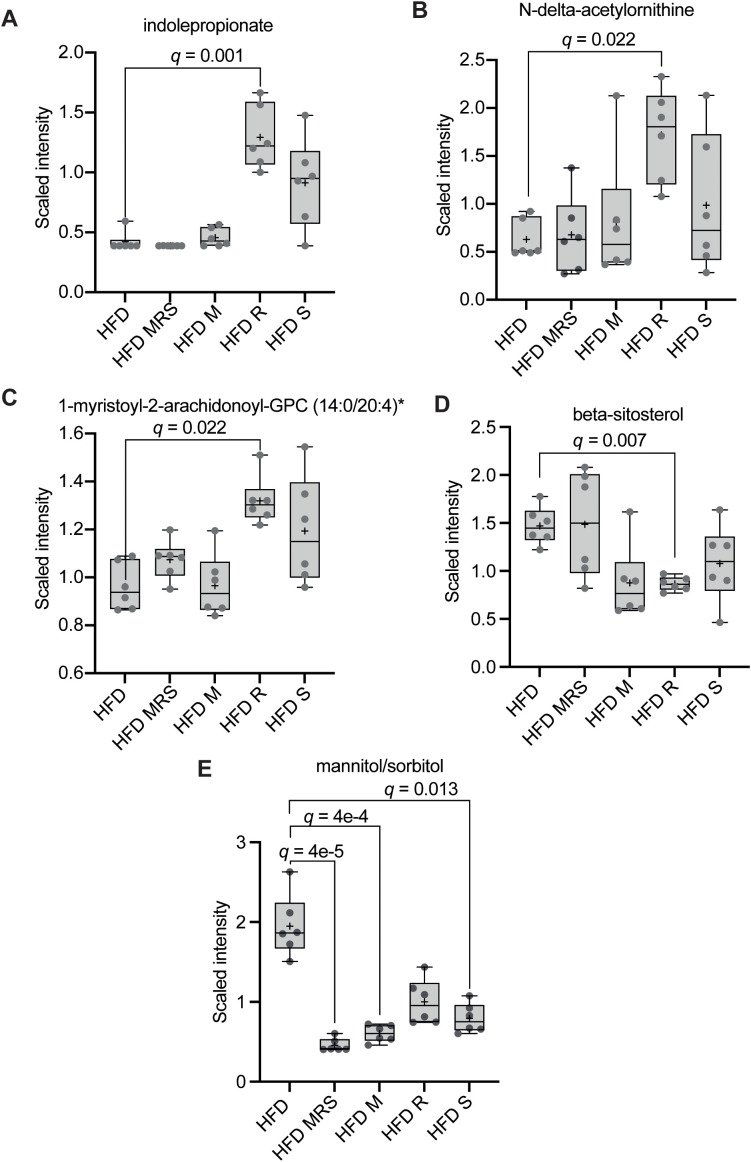
Effect of 2, 3-butanediol on the abundance of liver metabolites. **(A)** indolepropionate, **(B)** N-delta-acetylornithine, **(C)** 1-myristoyl-2-arachidonoyl-glycerophosphorylcholine (14:0/20:4)* [1-myristoyl-2-arachidonoyl-GPC (14:0/20:4)*], **(D)** beta-sitosterol and **(E)** mannitol/sorbitol. Male mice were fed a fat-enriched diet containing 16% fat (HFD). Cohorts were either untreated (HFD) or treated with 2, 3-butanediol isomer mix (HFD MRS), meso-2, 3-butanediol (HFD M), 2R, 3R-butanediol (HFD R), 2S, 3S-butanediol (HFD S). Mannitol and sorbitol are structural isomers and are not distinguishable by the analytical method used. All groups, n = 6.

To gain insights into the underlying causes of the effect of isomers of 2, 3-butanediol on weight gain we investigated the effect of the isomers on the concentrations of liver metabolites. Of the 693 metabolites identified, the concentrations of only four differed significantly (*q <* 0.05) between mice treated with the R isomer and untreated animals: indolepropionate, *N*-delta-acetylornithine and 1-myristoyl-2-arachidonoyl-glycerophosphorylcholine (14:0/20:4) being increased, and beta-sitosterol being decreased, in response to treatment with the R isomer (**Supplementary Table 1**; **Figures 2A–D**). Treatment of mice with the M (**Supplementary Table 2**) or S (**Supplementary Table 3**) isomers or with the mix of MRS (**Supplementary Table 4**) isomers had no significant effect on the concentration of any of these metabolites (**Figures 2A–D**). However, the M and S isomers and the MRS isomer mix significantly decreased the concentration of mannitol/sorbitol (**Figure 2E**; **Supplementary Tables 2**–**4**). Although the R isomer decreased the concentration of mannitol/sorbitol the effect was not significant (*q* = 0.060) (**Figure 2E**; **Supplementary Table 1**).

### Effect of treatment with isomers of 2, 3-butanediol on the gut microbiota of mice fed a fat-enriched diet

**Figure 3:**
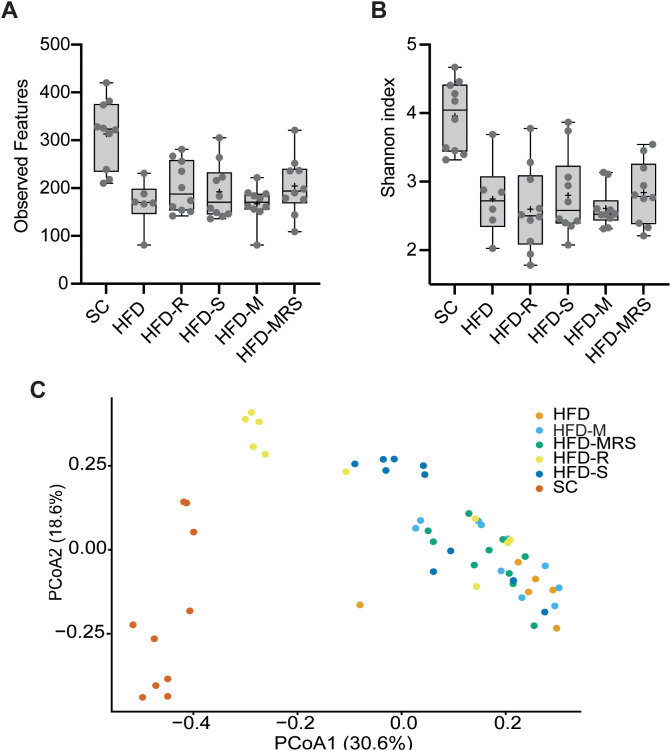
Changes in alpha- and beta-diversity of gut microbiota of mice in response to a fat-enriched diet (HFD) and to treatment with isomers of 2, 3-butanediol. **(A)** alpha diversity (observed features), **(B)** alpha diversity (Shannon index), **(C)** beta diversity (Bray-Curtis). Male mice were fed either a standard chow diet (SC) or a fat-enriched diet containing 16% fat (HFD). HFD cohorts were either untreated (HFD) or treated with 2, 3-butanediol isomer mix (HFD MRS), meso-2, 3-butanediol (HFD M), 2R, 3R-butanediol (HFD R), 2S, 3S-butanediol (HFD S). SC group, n = 10, HFD untreated, n = 7, HFD-treated cohorts, n = 10.

Indolepropionate, the liver metabolite whose concentration was most affected by treatment of mice with the R isomer of 2, 3-butanediol, is a product of gut bacteria (Roager and Licht, 2018). Consequently, we investigated the effect of 2, 3-butanediol isomers on the gut microbial composition of mice fed the HFD. Alpha-diversity analysis showed that the HFD reduced the overall richness of microbial species compared with that of mice fed the SC diet (*q* < 0.01) (**Figures 3A, B**), resulting in a significant difference in the diversity of gut bacteria between mice fed on the different diets. The effect of the HFD on the diversity of gut microbiota was not reversed by any of the 2, 3-butanediol isomers (**Figures 3A, B**). Beta-diversity analysis, using Bray-Curtis dissimilarity, showed a clear separation (PERMANOVA, *p* < 0.001) between the gut microbiota of mice fed the HFD or SC diet (**Figure 3C**), indicating a marked change in gut microbiota composition in response to the HFD. Although there was no complete separation between the untreated HFD group and any of the isomer-treated HFD groups, substantial subsets of both the R- and S-isomer groups clustered apart from the untreated HFD group (**Figure 3C**), suggesting that some of the mice in the R- and S-treated groups differed in beta-diversity from those in the untreated HFD group.

**Figure 4:**
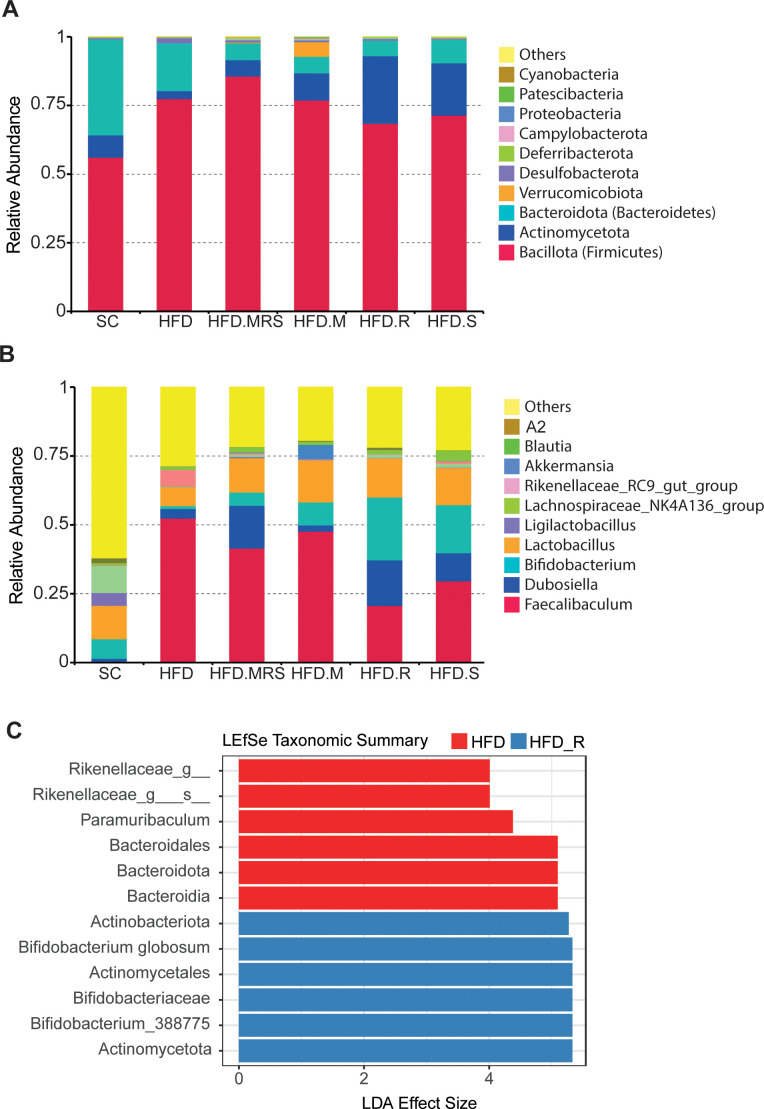
Effect of diet and 2, 3-butanediol isomers on the abundance of gut microbial taxa. Relative abundance of top 10 phyla **(A)** and top 10 genera **(B)** in gut microbiota of mice fed a standard chow diet (SC) or a fat-enriched diet containing 16% fat (HFD). HFD cohorts were either untreated (HFD) or treated with 2, 3-butanediol isomer mix (HFD MRS), meso-2, 3-butanediol (HFD M), 2R, 3R-butanediol (HFD R), 2S, 3S-butanediol (HFD S). SC, n = 10, HFD untreated, n = 7, HFD-treated cohorts, n = 10. **(C)** LEfSe taxonomic summary of HFD-fed mice either untreated (HFD) or treated with 2R, 3R-butanediol (HFD R), groups, n = 10.

At the phylum level, in comparison with the SC diet the HFD increased the proportion of Bacillota (Firmicutes) and decreased the proportions of Bacteroidota (Bacteroidetes) and Actinomycetota in the gut microbiota (**Figure 4A**). An increase in the ratio of Bacillota to Bacteroidota has been associated with obesity (Ley et al., 2005; Turnbaugh et al., 2009). Treatment of mice fed the HFD with the R or S isomer, and to a lesser extent the M isomer, increased the proportion of Actinomycetota and decreased that of Bacillota (**Figure 4A**). However, the MRS isomer mix did not decrease the proportion of Bacillota and had little effect on that of Actinomycetota (**Figure 4A**).

The lower microbial diversity in the gut of mice fed the HFD compared with those fed the SC diet (**Figures 3A, B**) suggests that compositional changes at the genus level would be more important. At the genus level, the HFD markedly increased the proportion of *Faecalobaculum* and decreased that of *Bifidobacterium* (**Figure 4B**). Treatment of mice fed the HFD with the R or S isomers decreased the proportion of *Faecalibaculum* and increased the proportion of *Bifidobacterium* (**Figure 4B**), thus partially reversing the effect of the HFD on the proportion of these bacterial genera in the gut. In contrast, the M isomer and the MRS isomer mix had little effect on the proportions of *Faecalibaculum* and *Bifidobacterium*. *Faecalibaculum* is a member of the Bacillota phylum and *Bifidobacterium* is a member of the Actinomycetota phylum. Thus, the changes in the proportion of these genera in response to the HFD and to treatment with the R or S isomers may account for the changes in the proportions of Bacillota and Actinomycetota at the phylum level.

LefSe analysis revealed significant differences in abundance of gut microbial taxa between untreated mice fed the HFD and those treated with the R isomer (**Figure 4C**). In comparison with that of treated mice, the gut microbiota of untreated mice was enriched in the Bacteroidota (Bacteroidetes) phylum, specifically in members of the Bacteroidia class, Bacteroidales order and Rikenellaceeae family (**Figure 4C**). Also enriched at the genus level was *Paramuribaculum*, a member of the Muribaculaceae family of the Bacteroidota phylum. In comparison with untreated mice fed the HFD, the gut microbiota of mice treated with the R isomer was enriched in the Actinomycetota phylum, specifically in the Actinomycetia class and Actinomycetales order. Also enriched were two species of *Bifidobacterium*, *B. globosum* and *B. 388775*, which are members of the Bifidobacteriaceae family and Bifidobacteriales order of the Actinomycetota phylum (**Figure 4C**).

## Discussion

Our results show that treatment of mice fed a fat-enriched diet (HFD) with a mix of 2, 3-butanediol isomers (MRS) had no effect on weight gain by the end of the seven-week treatment period, which is consistent with our previous finding that treatment for either 4 or 14 weeks with the butanediol isomer mix had no effect on weight gain of mice fed a SC diet (Veeravalli et al., 2022). In contrast, treatment for seven weeks with individual isomers of 2, 3-butanediol reduced weight gain of mice fed the HFD. The R isomer was particularly effective, totally preventing weight gain, thus acting as an anti-obesogenic agent. Although treatment with the R isomer prevented weight gain, it did not decrease weight.

The lack of effect of treatment with the butanediol isomer mix (MRS) on the plasma concentration of TAGs and NEFAs of mice fed the HFD is consistent with our finding that long-term treatment (14 weeks) with the isomer mix had no effect on the plasma concentrations of TAGs and NEFAs of mice fed a SC diet (Veeravalli et al., 2022). However, short-term treatment (4 weeks) with the isomer mix of mice fed a SC diet decreased the plasma concentrations of TAGs and NEFAs (Veeravalli et al., 2022). In contrast, treatment of mice fed the HFD with either the R or S isomers increased the plasma concentration of NEFAs.

The effect of butanediol treatment on the plasma concentrations of cholesterol and glucose of mice fed the HFD differed from those of mice fed a SC diet (Veeravalli et al., 2022). In mice fed the HFD, the plasma concentration of cholesterol was unaffected by the treatment with the isomer mix (MRS) or with any of the individual isomers, but in mice fed a SC diet plasma cholesterol was decreased by treatment with the isomer mix for either 4 or 14 weeks (Veeravalli et al., 2022). In mice fed the HFD, treatment with the isomer mix (MRS) or with the R or S isomers decreased the plasma concentration of glucose, whereas treatment of mice fed a SC diet with the isomer mix had no effect on plasma glucose (Veeravalli et al., 2022).

The decrease in the plasma concentration of glucose in mice fed the HFD elicited by treatment with the R isomer of butanediol would be expected to protect against diet-induced diabetes. But the increase in plasma concentration of NEFA in response to treatment with the R isomer would likely have the opposite effect, increasing the likelihood of developing insulin resistance (Eckel et al., 2005). However, the increase in NEFA may have been a compensatory effect, in response to the lower concentration of plasma glucose (Dimitriadis et al., 2021).

Indolepropionate, one of the three liver metabolites whose concentrations were significantly increased by R-isomer treatment of mice fed the HFD, is a gut microbiota-derived tryptophan metabolite which has advantageous effects on metabolic health and weight control (Roager and Licht, 2018). In rats, indolepropionate decreases fasting plasma glucose and insulin concentrations and increases insulin sensitivity (Abildgaard et al., 2018). In humans, indolepropionate is associated with a decreased risk of type-2 diabetes (De Mello et al., 2017), and lower concentrations of the metabolite are present in serum of obese females (Ruebel et al., 2019) and in plasma of patients with dyslipidaemia (Du et al., 2021). Thus, the effect of the R isomer of butanediol in preventing weight gain and decreasing the plasma glucose concentration of mice fed the HFD may be due, in part, to its ability to increase the concentration of indolepropionate in the liver.

Indolepropionate also has antioxidant and anti-inflammatory properties (Negatu et al., 2020) and reduces the risk of non-alcoholic fatty liver disease (Sehgal et al., 2022), and individuals with liver fibrosis have lower plasma concentrations of indolepropionate (Sehgal et al., 2021), indicating a potential hepatoprotective effect. A deficiency of indolepropionate is causally related to cardiovascular disease, and the molecule inhibits atherosclerosis by promoting reverse cholesterol transport (Xue et al., 2022).

In addition to indolepropionate, two other metabolites were increased in the liver of mice treated with the R isomer and both have been reported to have beneficial effects on human health. *N*-delta-acetylornithine is derived from the metabolism of arginine by bacteria such as *Bifidobacterium* (Nealon et al., 2019), and higher plasma concentrations are associated with lower risk of cardiovascular mortality and stroke (Smith et al., 2020). The phosphatidylcholine 1-myristoyl-2-arachidonoyl-glycerophosphocholine (14:0/20:4) is increased in the plasma of patients undergoing faecal microbiota transplantation treatment and is linked to pancreatic beta-cell preservation in recent-onset type-1 diabetes (De Groot et al., 2021).

The only molecule decreased in the livers of mice treated with the R isomer is beta-sitosterol, a phytosterol whose chemical structure resembles that of cholesterol (Babu and Jayaraman, 2020). It is a dietary component and occurs naturally in several foodstuffs. Mannitol/sorbitol, also a dietary-derived component, is decreased in the liver of mice fed the HFD in response to treatment with the M and S isomers and the MRS isomer mix. The HFD was fed to all butanediol isomer-treated cohorts, suggesting that the R isomer may have affected absorption of beta-sitosterol and the M and S isomers absorption of mannitol/sorbitol.

The decrease in the richness of the gut microbiota in response to the HFD is consistent with previous findings (Mamun et al., 2025) and has been associated with obesity, insulin resistance, inflammation and dyslipidaemia (Le Chatelier et al., 2013). None of the 2, 3-butanediol isomers had a significant effect on the alpha-diversity of the gut microbiota, but both the R and S isomers had partial effects on beta-diversity. Mice were fed the HFD for 10 weeks before being treated with 2, 3-butanediol isomers, leading to substantial changes in gut microbiota before the animals were exposed to the isomers. Therefore, it may not have been possible for subsequent treatment with the isomers to fully reverse the changes in the composition of the microbiota established in response to long-term feeding with the HFD and the lack of pre-treatment sampling of the microbiome is a limitation of the work.

One of the main effects of the R isomer on the gut microbiota of mice fed the HFD is the increase in the abundance of *Bifidobacterium* species, which are characteristic components of a beneficial microbiota. In both rodents (Waldram et al., 2009) and humans (Schwiertz et al., 2010) the abundance of *Bifidobacterium* is lower in obese than in lean individuals, and in humans it is negatively correlated with visceral fat area (Gong et al., 2022). Supplementation of *Bifidobacterium* to high-fat diet-induced obese mice suppressed weight gain and epididymal fat accumulation and decreased plasma glucose and insulin concentrations (Kondo et al., 2010), and the administration of *Bifidobacterium* species as probiotics to obese humans reduced body fat mass, body fat percentage and visceral fat area (reviewed in (Hamed Riveros et al., 2024)). Thus, the effect of the R isomer of butanediol in preventing weight gain and decreasing the plasma glucose concentration of mice fed the HFD may be due, in part, to its ability to increase the proportion of *Bifidobacterium* species in the gut microbiota by potentially modifying the gut environment.

Treatment with the R isomer did not increase the abundance of *Clostridium* or *Peptostreptococcus* species of the Bacillota phylum that are known to synthesize indolepropionate (Jiang et al., 2023). However, some *Bifidobacterium* species can produce indole-3-lactate (Aragozzini et al., 1979; Sakurai et al., 2019), which can be converted to indolepropionate by gut microbes (Zünd et al., 2025). Thus, the increase in the abundance of *Bifidobacterium* species in response to treatment with the R isomer may contribute to the increase in the concentration of indolepropionate in the liver.

Although each of the individual isomers of 2, 3-butanediol reduced weight gain of mice fed a high-fat diet, their effect was not additive, as the isomer mix (MRS) had little or no effect on weight gain. Despite the presence of the R isomer in the isomer mix (MRS), the MRS mix did not increase the concentration of indolepropionate in liver (**Figure 2A**) and had little effect on the proportion of *Bifidobacterium* in the gut microbiota. If the ability of the R isomer to prevent weight gain is mediated by increases in *Bifidobacterium* and indolepropionate, the lack of effect of the isomer mix on this bacterial genus and microbially derived liver metabolite may account for the inability of the isomer mix to prevent weight gain. It is possible that the higher total concentration of butanediol in the isomer mix had an inhibitory effect or that individual isomers had antagonistic effects.

The effects of the R isomer of butanediol in preventing weight gain and decreasing plasma glucose concentration in wild-type mice fed the HFD are similar to those of disruption of the *Fmo5* gene (Scott et al., 2017), but their mode of action differs. In the case of *Fmo5^-/-^* mice, disruption of the *Fmo5* gene has profound effects on liver metabolites, with the concentrations of more than 240 differing significantly (*q* < 0.05) from those in wild-type mice (Phillips et al., 2023). However, the concentrations of the three metabolites increased in response to treatment with the R isomer, indolepropionate, *N*-delta-acetylornithine and 1-myristoyl-2-arachidonoyl-glycerophosphorylcholine (14:0/20:4), were not increased in the liver of *Fmo5^-/-^* mice (Phillips et al., 2023). In addition, in contrast to the effect of the R isomer, the proportion of members of the *Bifidobacteriaceae* family in gut microbiota is not higher in *Fmo5^-/-^* mice fed a high-fat diet than in wild-type mice fed a high-fat diet (Scott et al., 2017). Thus the R isomer of 2, 3-butanediol appears to act via modification of the gut microbiota and production of microbially derived indolepropionate, whereas the effect of disruption of the *Fmo5* gene is independent of the gut microbiota (Scott et al., 2017).

## Conclusions

In the present study, we identified the R isomer of butanediol as an effective anti-obesogenic agent that prevented weight gain when wild-type mice are fed a 16% fat-enriched diet. The isomer also decreased the plasma concentration of glucose. Metabolomic studies of the liver showed that the isomer increased the concentration of indolepropionate, a microbially derived tryptophan metabolite which has been found to decrease plasma glucose and play a role in weight control in both rodents and humans. Analysis of the gut microbiota of mice fed a fat-enriched diet found that the 2R, 3R-butanediol increased the abundance of *Bifidobacterium* species, some of which can produce indole-3-lactate, which can be converted to indolepropionate by gut microbes. *Bifidobacterium* is considered to have beneficial effects: in mice it suppresses weight gain and fat accumulation and decreases plasma glucose; in humans it reduces fat mass. Thus, the effect of the R isomer of butanediol in preventing weight gain and decreasing the plasma glucose concentration of mice fed the fat-enriched diet may be due, in part, to its ability to increase the concentration of indolepropionate in the liver and the abundance of *Bifidobacterium* in the gut. Our results suggest that the R isomer of butanediol may be of value as a therapeutic agent to suppress weight gain in humans and its presence in several foodstuffs, including corn products (Buttery et al., 1994), fermented soy bean curds (Chung, 1999) and some fermented cocoa beans (Moreira et al., 2018), indicates that its use should be relatively safe.

## Data Availability

The microbial datasets presented in this study can be found in the ENA database under PRJEB89512. The metabolomic datasets are given in the **Supplementary Tables**.
